# *Salmonella* Typhimurium Level in Mealworms (*Tenebrio molitor*) After Exposure to Contaminated Substrate

**DOI:** 10.3389/fmicb.2020.01613

**Published:** 2020-07-23

**Authors:** Annette Nygaard Jensen, Sussie Hjort Hansen, Dorte Lau Baggesen

**Affiliations:** Division of Microbiology and Production, National Food Institute, Technical University of Denmark, Kongens Lyngby, Denmark

**Keywords:** *Tenebrio molitor*, *Salmonella*, contamination level, persistence, food safety

## Abstract

Findings of viable *Salmonella* spp., which are important foodborne pathogens, are seemingly not reported in mealworms (*Tenebrio molitor*) for feed and food. Still, the bacterial load of mealworms is naturally high and includes members of the Enterobacteriaceae family to which *Salmonella* belong. This indicates that *Salmonella* may be able to thrive in mealworms if introduced into the production. Therefore, this study aimed to assess the quantitative level of *Salmonella enterica* serovar Typhimurium (ST) in mealworms over a 14-day course after exposure to substrate contaminated with ST levels from 1.7 to 7.4 log CFU/g at start (i.e., day 0). The level of ST found in larvae was below the quantitative detection level (1 or 2 log CFU/g) on day 1 in larvae exposed to contamination levels of 1.7, 3.4, and 3.6 log CFU/g opposed to contamination levels of 5.4, 5.6, and 7.4 log CFU/g, respectively. The maximum level of ST detected in individual 1-g larvae samples was 5.8 log CFU/g, but the level varied among the triplicate samples from each sampling, and the highest average value was 5.3 ± 0.3. Beyond day 7, only larvae exposed to the contamination level of 7.4 log CFU/g were >1.0 log CFU/g in the triplicate samples. However, qualitative testing (10 g) showed the presence of ST in larvae until the end of the experiment on day 14 except for the lowest contamination level of 1.7 log CFU/g. Parallel testing of surface disinfected larvae indicated that some larvae may be ST-positive due to *Salmonella* residing on the surface only. Still, any detection of *Salmonella* is of concern from a food safety perspective. In substrate with contamination levels below 3.6 log CFU/g, the level of ST was below the quantitative detection limit within a few days. Still, ST was detected until the end of experiment on day 14 except for the lowest contamination level of 1.7 log CFU/g. This study indicates the importance of avoiding introduction of *Salmonella* into the production, e.g., via contaminated substrate in order to avoid *Salmonella-*positive larvae as they remained positive for at least 14 days (except at the lowest contamination level).

## Introduction

The interest in rearing of edible insects as a new valuable source of food and feed rich in proteins has increased significantly in the Western world following the publication ‘Edible insects – Future prospects for food and feed security’ from the Food and Agriculture Organization (FAO) in 2013 (van Huis et al., 2013). As for any other food and feed production chain, it is important to assess and control potential hazards associated to the production of insects and products derived thereof (Schlüter et al., 2016; van der Fels-Klerx et al., 2018; Raheem et al., 2019; Cappelli et al., 2020; Imathiu, 2020). Accordingly, The European Food Safety Authority (EFSA) prepared a risk profile related to production and consumption of insects as food and feed (EFSA Scientific Committee, 2015). Despite a scarcity of knowledge at that time about the specific risks associated to insects as a new form of mini-livestock, this risk profile emphasized the importance of hygienic conditions of the substrate and need for assessing the specific risks especially if other than food and feed-grade substrates are used (which is banned at present by EU regulation).

The EFSA risk profile also provided a list of insect species with high farming potential, which included the yellow mealworm (*Tenebrio molitor*) (EFSA Scientific Committee, 2015). As no appreciable consumption of mealworms took place in EU before May 15, 1997, mealworms are considered as Novel Foods by EU Regulation (EU) No 2015/2283 implying the requirement of authorization from EFSA before commercialization. Further, the general EU food law [Regulation (EC) No 178/2002] specifies that food and feed shall not be placed on market if unsafe. This also applies for insects even though the European Regulation (EC) No 2073/2005 on microbiological criteria for foodstuffs has set no specific criteria for mealworms (yet).

Due to a naturally high bacterial load of around 8 log CFU/g in *T. molitor* including opportunistic human pathogens, heat treatment or other processing is recommended to reduce the bacterial load before consumption (EFSA Scientific Committee, 2015; Schlüter et al., 2016; Stoops et al., 2016; Vandeweyer et al., 2017; Osimani et al., 2018; Wynants et al., 2018; Garofalo et al., 2019). The Enterobacteriaceae family is one of the highly represented bacterial groups in *T. molitor* larvae, and although *Salmonella* spp. belongs to this family, no findings of viable *Salmonella* has to our knowledge been reported yet (Grabowski and Klein, 2017; Vandeweyer et al., 2017; Osimani et al., 2018). This may be due to the use of feed-grade substrates as several insects as well as mealworms have vector potential for carrying *Salmonella* after exposure (Skov et al., 2004; Roche et al., 2009; Blazar et al., 2011; Nordentoft et al., 2017; Crippen et al., 2012, 2018; Wynants et al., 2019). Another explanation may be a potential capability of insects to fight incoming pathogens (Wu et al., 2018; Jo et al., 2019; Keshavarz et al., 2020).

As *Salmonella* spp. are important foodborne pathogens with >91,000 reported salmonellosis cases in EU annually, significant efforts are generally made to prevent their presence in feed and food production chains, e.g., stated by Regulation (EC) No 2160/2003 (European Food Safety Authority [EFSA], and European Centre for Disease Prevention, and Control [ECDC], 2019). A broad range of animals can carry *Salmonella* spp. and often without any symptoms, causing fecal excretion of *Salmonella* into the environment and potential transmission to other animals, crops and water reservoirs. Consequently, contaminated substrate, insufficient hygienic measures or lack of biosecurity for preventing entrance of infected wild insects, rodents and pets may introduce *Salmonella* spp. into insect production facilities.

For documenting the absence of *Salmonella* spp. in food, qualitative testing of the presence of *Salmonella* spp. by pre-enrichment of 10-g or 25-g samples is usually applied (e.g., ISO, 2017 6579-1). However, the level of *Salmonella* contamination is also of interest as dose–response modeling of *Salmonella* using outbreak data indicated that both the risk of infection and the risk of illness given infection increase with dose (Teunis et al., 2010). The dose response model found that the number of CFUs causing infection or illness in 50% (ID50) of exposed people were 7 and 36 CFU, respectively. Noteworthy, the bacterial cell count obtained from a sample, will depend on the ability to retrieve cells adequately from the sample matrix, which is affected by the nature of the matrix (e.g., fat content, physical structure) and the homogenization method applied (Rohde et al., 2015). Accordingly, proper sample treatment is important for enumerating the actual number of cells present and avoiding underestimation or false negative results. Still, there seem to be no suggestions for or evaluations of a proper and standardized treatment method for mealworm samples before enumeration or detection of the bacterial content.

One recent study assessed how the presence of *Salmonella* sp. during rearing of mealworms affected the survival in substrate and transmission to larvae (Wynants et al., 2019). The time course was here 7 days and it is of interest to see if longer time, e.g., will support clearance of *Salmonella*, which is relevant concerning mitigation options. In addition, more contamination levels, higher frequency of samplings and quantitative testing may help to a better understanding of the fate of *Salmonella* after introduction into a mealworm production site as well as the importance of the *Salmonella* contamination level.

In this study, we firstly aimed to test the ability to recover *Salmonella* Typhimurium from mealworm samples with the applied detection method. Secondly, we aimed to assess the quantitative level of *S.* Typhimurium in disinfected and non-disinfected mealworms over a 14-day course after exposure to substrate contaminated with different levels of *Salmonella* at start, as well as the fate of *Salmonella* in the substrate.

## Materials and Methods

### Mealworms

Mealworms (*Tenebrio molitor*, L.) provided by the Danish Technological Institute (Aarhus, DK) were reared in-house at the National Food Institute, Technological University of Denmark (DTU) (Kgs. Lyngby, DK) on a flour-based dry substrate provided by Adival A/S (Billund, DK) and spent grain acquired from DTU Brewery (Kgs. Lyngby, DK) as wet feed. The rearing room had a temperature of 26.5 ± 0.6°C and a relative humidity of 55.0 ± 3.6%.

### *Salmonella* Typhimurium Contamination Strain

A Danish strain of *Salmonella enterica* serovar Typhimurium DT12 (*S.* Typhimurium or ST) previously made resistant to rifampicin (Jensen et al., 2006) was used to contaminate the substrate in an experimental laboratory study. A colony from an overnight (o.n.) plate-spread on tryptic soy agar with sheep blood (TSASB) (98763, SSI Diagnostica, Copenhagen, Denmark) was transferred into 10 mL Buffered Peptone Water (CM1049, Oxoid, DK) and incubated o.n. at 37°C. A 10-fold dilution series of the o.n. culture was prepared in 0.9% NaCl solution to reach expected contamination levels ranging from approximately 2 to 7 log CFU/g, and 0.1 mL of each dilution was plate-spread onto TSASB to determine the exact concentration of ST.

### Quantitative Estimation of *S.* Typhimurium

To estimate the level of *S.* Typhimurium in larvae, 1 g of larvae was sampled and added 1 mL 0.9% NaCl solution before homogenization in-tube (12 mL round-bottomed tube) by grinding with a sterile Thomas pestle tissue grinder made of teflon and with at stainless steel shaft (Thomas Scientific, Swedesboro, NJ, United States). Additional 8 mL saline was added to the tube (i.e., 10^–1^ dilution) before vortexing and preparation of a 10-fold dilution series in 0.9% NaCl. Appropriate dilutions were spread onto Nutrient Agar (NA) plates with 50 μg/mL rifampicin (R3501, Sigma-Aldrich Chemie, DE) (NA^rif^). For samples expected to have concentrations <100 CFU/g, 1 mL of the 10^–1^ dilution was distributed onto three agar plates to reach a detection limit of 10 CFU/g, otherwise 0.1 mL was plated.

Substrate samples of 1 g were handled similarly, except that the homogenization step was performed simply by vortexing 15 s.

The agar plates were incubated o.n. at 37°C before enumeration of ST presumptive colonies. Suspect colonies were verified by sub-culturing onto indicative plates as well as NA and NA^rif^ plates and agglutination serotyping as described in Section “Qualitative Detection of *S.* Typhimurium.”

### Qualitative Detection of *S.* Typhimurium

Qualitative detection of *S.* Typhimurium in homogenized larvae and substrate samples (10 g) was done by addition of 90 mL of buffered peptone water (BPW) (94515, SSI Diagnostica) before o.n. pre-enrichment at 37°C. The 10 g larvae was homogenized by grinding with a pestle tissue grinder as above, but in a 50 mL tube added 2 mL BPW, and with transfer of the larvae material to a 150 mL cup by flushing the tube with additional 88 mL BPW in total. The o.n. culture was spotted onto modified semi-solid Rappaport–Vassiliadis (MSRV) agar (CM0910 and SR0161, Oxoid, DK) in three droplets (approx. 0.1 mL). After o.n. incubation at 41.5°C, material from presumptive *Salmonella* swarming zones was sub-cultured onto indicative plates Brilliant Green Agar plates (PO5033A, Oxoid) and Xylose Lysine Deoxycholate (XLD) agar plates (PO5057A, Oxoid) and incubated at 37°C o.n.

The ST contamination strain was verified by slide agglutination of colony material from the indicative plates with O4 (23839, SSI Diagnostica, DK) and O5 (40272, SSI Diagnostica) antiserum according to the Kauffmann–White scheme (Grimont and Weill, 2007) as well as confirmation of the resistance marker by sub-culturing onto NA and NA^rif^.

### Detection Limit for *S.* Typhimurium in Mealworms

The ability to detect *Salmonella* in mealworms was assessed by testing larvae artificially contaminated with specified levels of *S.* Typhimurium. A ST suspension (in duplicate) was prepared as described in Section “*Salmonella* Typhimurium Contamination Strain” and diluted tenfold. Then 100 μl of each dilution was added to 1-g larvae samples to reach *Salmonella* concentrations from 1 to 10^5^ CFU/g larvae. The added cells were allowed to settle on the larvae for 10 min, while kept at 5°C to avoid growth of the added cells and to calm the larvae before quantification of *Salmonella* as described in Section “Quantitative Estimation of *S.* Typhimurium.”

Additional, qualitative detection of *Salmonella* was performed as described in Section “Qualitative Detection of *S.* Typhimurium.” Larvae samples of 10 g were added ST in numbers from approximate <1 to 100 CFU from appropriate dilutions of the suspension (100 μl was added).

### Experimental Exposure of Mealworms to *Salmonella*-Contaminated Substrate

In two successive experiments, 50–60 days old larvae close to the stage of pupation were exposed to substrate experimentally contaminated with different concentrations of *S.* Typhimurium at start of the experiment. Each experimental period lasted for 14 days. The experimental trays with larvae were placed in a separate incubator in the laboratory to avoid accidental contamination of the rearing room at temperatures of 25.5 ± 0.3°C and 25.6 ± 0.1°C and a RH of 55.9 ± 6.1% and 39.6 ± 4.6% in trials 1 and 2, respectively.

#### Experimental Trial 1

For the experimental set-up, larvae with an average weight of 126 ± 20 mg were sieved to remove the substrate provided during rearing. Two days prior to exposure on day zero, larvae, and fresh flour-based dry substrate (Adival A/S) were allocated in a 1:1 ratio of 150 g into each of 4 aluminum foil trays (19 cm × 12.5 cm × 4.5 cm, 1.1 L). One additional tray was added substrate only (150 g).

On day zero, 1.7 mL of appropriate dilutions of the *S.* Typhimurium suspension was added to 50 mL Greiner tubes each containing 20 g spent grain (DTU Brewery). After thorough vortexing, the contaminated spent grain was transferred to the aluminum foil trays with larvae for estimated final ST concentrations of approximately 2, 4, and 6 log CFU per g of substrate in each tray, respectively, see Table 1. Additionally, contaminated spent grain was added to the tray with substrate only (4 log CFU/g of substrate) referred to as control, while 20 g uncontaminated spent grain was added to a tray with larvae as a negative control for cross-contamination between trays. During the experimental period, the larvae were provided water by addition of 20 g spent grain after each sampling.

**TABLE 1:** Set-up for experimental exposure of mealworm to *Salmonella*- contaminated substrate.

	*Salmonella* contamination level (expected log CFU/g)^a^			
	
Tray	Description	1st trial	2nd trial	Tray content^b^
1	Low	2	3	Larvae: substrate (1:1)
2	Medium	4	5	Larvae: substrate (1:1)
3	High	6	7	Larvae: substrate (1:1)
4	Neg. control	0	0	Larvae: substrate (1:1)
5	Control	4	5	Substrate

**^*a*^Appropriate dilutions of the S. Typhimurium suspension were added via 20 g spent grain to reach an ‘expected’ final contamination level in the substrate at start of the experiment. ^*b*^Larvae were mixed with fresh dry substrate in a 1:1 ratio of 150 g 2 days prior to contamination with Salmonella*.*

#### Experimental Trial 2

The set-up was similar to the first trial but with the following modifications. The average larvae weight was 131 ± 9 mg. The fixed volume the of *S.* Typhimurium suspension used to contaminate the spent grain was reduced to 1 mL and the contamination level was based on the results of the first trial increased to estimated final ST concentrations of approximately 3, 5, and 7 log CFU per g of substrate, respectively, see Table 1. The control tray without larvae was added 5 log CFU per g of substrate.

#### Sampling of Larvae and Substrate

The first sampling was performed approximately 10 min after addition of spent grain, referred to as day 0. During each experimental period, samplings were performed on day 0, 1, 2, 5, 7, 9, 12, and 14 or until a sample rendered test-negative by qualitative testing. The negative controls were sampled days 0, 7, and 14.

From each tray, 1 g of larvae was transferred to a round-bottomed 12 mL tube with a sterile tweezer (*n* = 6) for quantitative detection of *S.* Typhimurium (see section “Quantitative Estimation of *S.* Typhimurium”). Half of the larvae samples from each sampling was surface disinfected before testing (see section “Surface Disinfection of Larvae”). Following a negative test result by the quantitative testing approach, 10 g of larvae was transferred to a 50 mL Greiner tube for qualitative testing for the presence of ST (see section “Qualitative Detection of *S.* Typhimurium”).

Substrate samples of 1 g mainly consisting of the flour-based substrate (leaving out spent grain residues) were collected from each tray with a plastic spoon and transferred to a 50 mL Greiner tube (*n* = 3) for quantitative detection of ST (see section “Quantitative Estimation of *S.* Typhimurium”). As for larvae, a 10-g sample was collected for qualitative testing when the preceding sample was test negative.

#### Surface Disinfection of Larvae

To remove *Salmonella* present on the surface of larvae, 1 g larvae samples were collected into a tube and added 3 mL of 70% w/w ethanol and vortexed for 10 s before removal of the ethanol. The larvae were left for 2 min before being rinsed twice with 5 mL Milli Q water.

This method had been tested prior to this study (data not shown). Shortly, individual larva was submerged in a suspension of the ST strain (approx. 10^7^ CFU/mL), then 1 g larvae (*n* = 3) were pooled and disinfected by the method described above before individual larva was pour plated in 25 mL NA^rif^ and incubated overnight at 37°C.

### Statistical Analyses

The quantitative detection of ST in larvae was compared with the concentration of ST added (Table 2) by pairwise *t*-test in GraphPad Prism version 8.3.1 for Windows, GraphPad Software, San Diego, CA, United States, www.graphpad.com (RRID:SCR_002798). For substrate, ST counts for each contamination level were compared between sampling moments (Table 3) using repeated measures ANOVA in GraphPad Prism. When counts were below the detection limit applied, i.e., 1 or 2 log CFU/g depending on the expected count, a value representing half of the detection limit, i.e., 0.5 or 1.0 log CFU/g, respectively, was chosen as a value to be included in the statistical analysis. Qualitative testing was applied following a sampling with a ST count below the detection limit, where values of 0.0 and −0.3 were assigned for positive and negative results, respectively.

**TABLE 2:** Quantitative detection level for *S.* Typhimurium in mealworms.

ST in mealworm samples (log CFU/g)			

1st trial		2nd trial	
	
Cells added^a^	Cells detected	Cells added^a^	Cells detected
5.01	4.90	5.21	5.10
4.01	4.00	4.21	4.08
3.01	2.62	3.21	2.60
2.01	2.15^b^	2.21	2.20^b^
1.01	ND^c^	1.21	1.70^c^
0.01	ND^c^	0.21	ND^c^

**^*a*^Addition of 100 μL ST cell suspension (10-fold dilution series) to 1 g mealworm. ^*b*^Weighted average of CFU/g for plating of 100 μL (in duplicate) and 1 mL of the 10^–1^ dilution. ^*c*^Plating of 1 mL of the 10^–1^ dilution ND, none detected*.*

**TABLE 3:** Level of *Salmonella* Typhimurium found in substrate, larvae and disinfected larvae over a 14 days period after a single contamination event of substrate with ST at start.

Sample	*Salmonella* added^a^ (log CFU/g)	*S.* Typhimurium log CFU/g^b^							
		
		Day after contamination of substrate							
		
		0	1	2	5	7	9	12	14
Larvae disinfected	1.7	0.5 ± 0.9	<1.0	<1.0	–	–	–	–	–
	3.4	1.4 ± 1.2	1.1 ± 1.9	Neg	Pos	–	Pos	Neg	–
	3.6	2.2 ± 0.4	<2.0	<2.0	–	–	Neg	–	Neg
	5.4	3.8 ± 0.5	<2.0	1.6 ± 2.7	1.0 ± 1.7	<1.0	<1.0	Pos	Neg
	5.6	4.1 ± 0.3	<2.0	<2.0	0.7 ± 1.2	<2.0	Pos	<2.0	Neg
	7.4	5.3 ± 0.3	2.0 ± 1.8	2.2 ± 2.0	<1.0	2.3 ± 2.4	<2.0	<1.0	Pos
Larvae	Control	Neg	–	–	–	Neg	–	–	Neg
	1.7	0.8 ± 1.4	<1.0	Neg	Neg	Neg	–	–	–
	3.4	1.5 ± 0.5	<1.0	Neg	Pos	–	Pos	Pos	Pos
	3.6	3.0 ± 0.4	<2.0	Pos	Pos	Pos	Pos	Pos	Pos
	5.4	3.8 ± 1.3	2.4 ± 2.1	<2.0	2.1 ± 0.5	1.2 ± 2.1	<1.0	Pos	Pos
	5.6	5.0 ± 0.1	2.4 ± 2.2	2.0 ± 3.5	Pos	Pos	Pos	Pos	Pos
	7.4	5.2 ± 0.5	2.1 ± 1.8	<2.0	3.1 ± 0.5	3.1 ± 1.2	2.3 ± 2.2	2.3 ± 0.3	1.9 ± 0.3
Substrate with larvae	Control	Neg	–	–	–	Neg	–	–	Neg
	1.7	0.9 ± 1.5	0.7 ± 1.2	<1.0	–	Neg	–	–	–
	3.4	1.6 ± 1.4	<1.0	Neg	Pos	–	Pos	Pos	Pos
	3.6	2.5 ± 2.2	0.7 ± 1.2	<2.0	–	Pos	Pos	–	Pos
	5.4	4.1 ± 0.7^AC^	1.4 ± 1.2^BC^	<2.0^AB^	3.7 ± 0.4^AC^	3.2 ± 0.3^CD^	1.8 ± 0.5^BD^	2.5 ± 0.4^BCD^	2.3 ± 0.2^AB^
	5.6	4.7 ± 0.8^AB^	3.3 ± 0.5^A^	2.5 ± 0.4^B^	2.1 ± 1.8^AB^	3.0 ± 1.2^AB^	2.8 ± 0.8^B^	2.0 ± 2.0^AB^	2.0 ± 2.0^AB^
	7.4	6.0 ± 0.4^AC^	3.4 ± 0.2^AB^	<2.0^C^	3.8 ± 0.1^AB^	4.6 ± 0.5^AB^	3.7 ± 0.1^B^	3.5 ± 0.2^B^	3.7 ± 0.1^B^
Substrate without larvae	3.4	1.7 ± 1.5	<2.0	<2.0	–	Pos	Pos	Pos	Pos
	5.4	4.5 ± 0.5	<2.0	<2.0	0.4 ± 0.8	0.3 ± 0.6	Pos	Pos	Pos

**^*a*^Estimated concentration of S. Typhimurium in substrate after the contamination event on day 0; Concentrations of 1.7, 3.6, and 5.6 log CFU/g were added in trial 1 and 3.4, 5.4, and 7.4 log CFU/g were added in trial 2; no ST was added to the controls. ^*b*^Average values of triplicate samples ± standard deviation, with application of a value of zero for each individual sample negative in the quantitative testing. Triplicate samples all negative in the quantitative testing are indicated as below the applied detection limit, i.e., <1 or <2 log CFU/g. Detection of ST by qualitative testing (10-g samples) is shown as Pos while no detection of ST is shown as Neg; –, no testing; ST counts within each row that share a letter in superscript, did not significantly (p ≥ 0.05) increase of decrease between sampling days, as was shown from repeated measures ANOVA.**

## Results

### Detection Limit for *S.* Typhimurium in Mealworms

Colonies of the *S*. Typhimurium contamination strain on NA^rif^ agar plates were counted based on morphology of colonies verified to be the contamination strain as described in Section “Quantitative Estimation of *S.* Typhimurium” and Section “Qualitative Detection of *S.* Typhimurium.” The quantitative detection of the ST contamination strain, showed that the detection level was close or similar to the level of cells added (*p* = 0.45), see Table 2.

The added cell levels of 1.01 and 1.21 log CFU/g in trials 1 and 2, respectively, are close to the theoretical detection level of 1 log CFU/g when 1 mL of the 10^–1^ dilution is plated, which may explain the negative test-result obtained for 1.01 log CFU/g in trial 1.

For the qualitative detection method, *S.* Typhimurium was detected in 1 out of 2 larvae samples at a contamination level of 0.6 CFU per 10 g sample, while both sample replicates were positive when the contamination level was 10-fold higher, i.e., 6 CFU or higher.

### Experimental Exposure of Mealworms to *Salmonella*-Contaminated Substrate

Mealworms were at start of each of the two experimental periods exposed to substrate contaminated with *S.* Typhimurium in concentrations ranging from 1.7 to 7.4 log CFU/g. The quantitative level of ST found in larvae and substrate during the 14-day study period is shown as an average of triplicate samples (1 g each) in Table 3. In case individual samples tested negative, i.e., below the detection limit of 1 or 2 log CFU/g, a value of zero was applied for calculation of the average.

The level of ST found in larvae was below the quantitative detection level in all three samples (in Table 3 shown as <1 or <2 log CFU/g) already within 1 day in larvae exposed to contamination levels of 1.7, 3.4, and 3.6 log CFU/g opposed to contamination levels of 5.4, 5.6, and 7.4 log CFU/g, respectively. The maximum level of ST detected in individual 1-g larvae samples (i.e., 8 larvae) was 5.8 log CFU/g detected on day 0. Beyond day 7, only larvae exposed to the highest contamination level, i.e., 7.4 log CFU/g were >1.0 log CFU/g, but often with variation of the ST level in individual larvae samples with findings of, e.g., <2.0, 2.4, and 4.4 log CFU/g on day 9. At end of the experiment on day 14, the ST level in these larvae was 1.9 ± 0.3 log CFU/g.

When testing of 1-g larvae samples (non-disinfected) from each experimental tray reached the detection limit, 10-g samples were tested qualitatively for the presence of ST in the succeeding samplings. This showed the presence of ST in non-disinfected larvae at all contamination levels until the end of the experiment on day 14 as indicated by Pos for positive in Table 3 (i.e., at least 1 CFU/g) except for the lowest contamination level of 1.7 log CFU/g. Here, the ST level was <1.0 log CFU/g on day 1 and no ST was found in three succeeding samplings on days 2, 5, and 7.

In the negative control tray where no ST was added, weekly testing of larvae and substrate showed absence of ST, i.e., no indication of cross-contamination between the experimental trays.

In this experiment as well as under natural rearing conditions, mealworms inhabits their substrate and therefore surface disinfection was applied in an attempt to disclose whether detected *S.* Typhimurium derived from ingested ST or merely from contamination on the surface of the larvae. The applied disinfection method with 70% ethanol and washing twice in MilliQ water had been tested prior to this study (see section “Surface Disinfection of Larvae”), where visual inspection of the larvae embedded in the agar plates showed no growth of ST on 20 out of the 21 larvae disinfected in total. ST was found on a single larva but with markedly lower growth of ST than on control larvae rinsed with water only, overall indicating efficacy of the applied disinfection method.

For surface disinfected larvae, ST was on day 0 detected in 15 out of 18 larvae samples and not in levels markedly different from non-disinfected larvae. However, at later samplings, the disinfected larvae generally rendered test-negative earlier than the untreated larvae, and only at the highest contamination level of 7.4 log CFU/g was ST found present in disinfected larvae at the end of experiment on day 14 (see Table 3). The qualitative testing of disinfected larvae was performed less frequently not to skew the 1:1 ratio between substrate and larvae too much when removing samples of 10 g, and further, it was considered superfluous if the non-disinfected counterpart tested negative already.

In the substrate, the level of *S* Typhimurium on day 0 shortly after contamination was up to 1.4 log lower than the expected level of ST added (see Table 3). At the three lowest contamination levels (1.7–3.6 log CFU/g), the ST level in the substrate was below the detection limit on day 2 and onwards. Still, ST was found until the end of experiment by qualitative testing except for the low contamination level, i.e., 1.7 log CFU/g. At the contamination levels of 5.4, 5.6, and 7.4 log CFU/g, the level of ST detected in the substrate on day 14 was 2.3 ± 0.2, 2.0 ± 2.0, and 3.7 ± 0.1 log CFU/g, respectively.

The level of *S.* Typhimurium in substrate from trays without larvae (control) was compared with ST levels in the trays containing larvae at contamination levels of 3.6 and 5.4 log CFU/g in trials 1 and 2, respectively (see Table 3). In the first trial, ST was generally below the quantitative detection level within the first days both in trays with and without larvae present although ST was detected until the end of the experiment. At the higher contamination level in the second trial, the ST level in the substrate remained quantifiable in trays with larvae, while the substrate without larvae after day 7 was ST-positive by pre-enrichment only.

## Discussion

In order to assess the level of *Salmonella* Typhimurium cells in mealworm (*T. molitor*) larvae after exposure to contaminated substrate, we made a preliminary test of the ability to recover *S.* Typhimurium from mealworm samples with the applied detection method. A standard approach for determination of bacterial cell numbers in a sample is spreading of a sample dilution series on agar plates. This approach though, has a theoretically limit of detection depending on the portion of sample plated. Further, the high bacterial load generally present in mealworms may challenge the specific detection of the *S.* Typhimurium target strain if outcompeted by the inherent bacteria (Vandeweyer et al., 2017; Wynants et al., 2018). Therefore, a *S.* Typhimurium strain with an antibiotic resistance marker (rifampicin) previously proven adequate for detection in pig fecal samples (Jensen et al., 2006) was chosen for the study to facilitate detection. Other strains of S. Typhimurium or other serovars may not have elicited the exact same outcome, however, *S.* Typhimurium is one of the most important serovars in human cases of salmonellosis and was considered a good candidate for the exposure study as a start.

Proper retrieval of bacterial cells from a sample is also important to obtain a good recovery of the bacteria of interest (Rohde et al., 2015). In this study, in-tube homogenization of mealworm samples by grinding with a sterile pestle tissue grinder was applied, as a small preliminary study indicated a good recovery of cells by this method as compared to stomaching and crushing by hands. This preliminary evaluation was based on total aerobic count of the inherent bacterial population (data not shown), as artificial spiking with the bacteria of interest is unlikely to reflect the natural binding or embedment of cells within a sample matrix. The in-tube homogenization approach appears convenient opposed to, e.g., blending or use of a mortar as there is no need for transferring the sample material after homogenization. Moreover, such transfer may lead to loss of material if the sample is weighed beforehand or failure in achieving a fully representative sample if weighing is performed on a sample not completely homogenous, which is likely for mealworms and other insects due to their exoskeleton parts.

The preliminary assessment of the detection limit for *S.* Typhimurium in mealworm samples indicated a good recovery of the cells added both for the quantitative estimation (Table 2) and the qualitative detection method applied in this study. The level of ST added in trial 1 was estimated to 1.01 log CFU/g, i.e., very close to the theoretical detection limit of 1 log CFU/g when 1 mL of the 1:9 (10^–1^) dilution is plated. So the lack of detection at this level seemed to be within the expected precision of the plate spreading method rather than suppression of ST growth, generally implying a low risk of incorrect conclusions due to false-negative test results.

In the experimental exposure study, the lowest contamination level of 1.7 log CFU/g was the only one not resulting in ST positive mealworms at the end of the experiment, i.e., on day 14 for the non-disinfected larvae. In a study by Wynants et al. (2019), a *Salmonella* contamination level of 2 log CFU/g also resulted in test-negative larvae on day 7 where the experiment ended. At a contamination level of 4 log CFU/g, those authors found 4 of 6 larvae replicates *Salmonella*-negative (qualitative testing only) on day 7 in one experiment, while *Salmonella* was <1.0 log CFU/g in another experiment. Similarly, in the current study, contamination levels slightly lower at 3.4 and 3.6 log CFU/g resulted in *Salmonella*<1.0 log CFU/g and <2.0 log CFU/g on day 1. A detection limit of 2 log CFU/g was applied for samples expected to reach this level, but the obtained ST counts proved to be lower in some cases, and here a detection limit of 1 log CFU/g would have been more informative. Nevertheless, the non-disinfected larvae remained ST positive throughout the experimental period of 14 days. Even though this may imply a *Salmonella* presence as low as 1 CFU per gram, the ID50 of 7 CFU for causing infection emphasizes the significance of these findings (Teunis et al., 2010). It also supports the recommendations concerning heat treatment or other processing to ensure the food safety of mealworms, although no specific food safety criteria [Regulation (EC) No 2073/2005] for edible insects have been established (yet).

The surface disinfected larvae generally turned ST-negative somewhat earlier than the corresponding non-disinfected larvae, indicating that some ST resided on the surface only. Surprisingly though on day 0, the ST levels were similar in the disinfected and non-disinfected larvae. Given efficacy of the disinfection method, this implies a rapid ingestion of substrate (*Salmonella*), as sample collection were started approximately 10 min after addition of the contaminated spent grain. To our knowledge, no other studies have made a parallel testing of disinfected and non-disinfected larvae in the same experiment to shed light on this. Inefficient disinfection could also explain the high ST counts on day 0, and despite our promising pre-evaluation of the method based on 70% ethanol, results by Crippen and Sheffield (2006) indicated that 70% ethanol alone was inefficient for surface disinfection of beetles of the lesser mealworm (*Alphitobius diaperinus*). However, for their assessment, disinfected beetles were submerged completely into the growth medium, and although shortly, excretion of internal bacteria into the medium during the submersion cannot be excluded. Regardless, any detection of *Salmonella* either internal or external is of concern from a food safety perspective, as the whole larvae will be processed.

At a contamination level of 7 log CFU/g, Wynants et al. (2019) observed no decrease in the *Salmonella* level in larvae on day 7 (4.1 ± 1.1 log CFU/g) and it was discussed whether it was simply a matter of longer time needed to exert a reduction or if numbers were too abundant for a reduction to happen. The only other contamination level tested in that experiment was 4 log CFU/g, which resulted in <1 log CFU/g in larvae at day 7, i.e., the fate of *Salmonella* at contaminations between 4 and 7 log CFU/g is uncertain. In our study, the ST counts for 7.4 log CFU/g were 5.2 ± 0.5, 3.1 ± 1.2, and 1.9 ± 0.3 log CFU/g on days 0, 7, and 14, respectively and at 5.4 log CFU/g, the ST counts were <1.0 log CFU/g on day 9 but ST positive until day 14. This indicates a decrease over time but also that the additional 7 days in the current study were insufficient for clearing the contamination in the larvae. Also Crippen et al. (2012) found that the lesser mealworm excreted *Salmonella* into their feces (frass) for 6–12 days after a 2 h exposure to 8 log CFU/mL, and where 33 and <10% of the larvae shed *Salmonella* on day 9 and day 12, respectively. In that study, however, the larvae were isolated from the source of contamination after 2 h, i.e., not reflecting real life rearing conditions where larvae inhabit their substrate, where contaminations may persist and constitute a continuous exposure. Although the ST counts found in our larvae depended on the initial contamination level, absence of ST within the 14-day course was not observed in non-disinfected larvae for contaminations levels ≥3.4 log CFU/g. So one can still pose the questions whether longer time (>14 days) was needed to clear the contamination in the larvae or if there is a certain threshold (>2 log CFU/g based on our study and Wynants et al., 2019) above which *Salmonella* will persist until harvest prior to pupation. Further, the answers may depend on several other factors like type of substrate, larval density or the larval stage at which the contamination event occurs, as younger larvae may elicit less colonization resistance to foreign microorganisms (Wynants et al., 2019). Moreover, it is uncertain how the design of the experimental set-up affects the results. For example, the volume of *Salmonella* suspension chosen for contaminating the substrate will affect the moisture content and hence growth potential of bacteria, while the method of distribution may influence the actual level of contamination experienced by individual larvae.

Concerning the current experimental design, the ST was added via the wet substrate (spent grain) to avoid clumping of the dry flour-based substrate. The spent grain constituted 12% of the substrate in total, and despite mixing efforts it may have caused an uneven distribution of ST and partly explain the discrepancy (up to 1.4 log) between the ST counts obtained on day 0 and the ST numbers added (Table 3). However, *Salmonella* contaminations occurring under natural rearing conditions will most likely result in a heterogeneous distribution of cells as well, and although larvae activity probably aid a more homogeneous distribution of contaminants over time, the triplicate substrate and larvae samples had variable ST counts as evident from the standard deviations of the averages. This possible variation between samples will also need consideration when sampling for monitoring purposes.

In substrate contaminated at levels ≤3.6 log CFU/g, ST counts were soon below the detection limit, although only ST-negative on day 14 for the lowest contamination level (1.7 log CFU/g) as the case for the larvae. At the 5.4 and 5.6 log CFU/g contamination levels, the ST counts in substrate were reduced to 2.3 ± 0.2 and 2.0 ± 2.0 on day 14, respectively, but counts were not significantly different from the initial ST level counted on day 0 (Table 3). Noticeable, although the ST contamination level found in substrate on day 14 in these trays was close to the initial contamination level of 1.7 log CFU/g where larvae samples rendered ST-negative within the first days, the larvae in these trays were still ST-positive on day 14. Similarly, the initial 7.4 log CFU/g contamination level in substrate decreased significantly to 3.7 ± 0.1 log CFU/g on day 14, but this level was sufficient for ST counts reaching 1.9 ± 0.3 log CFU/g in the larvae opposed to trays with initial contamination levels of 3.4 and 3.6 log CFU/g. All together indicating the importance of the initial contamination level in the substrate for the resulting ST counts in the larvae. Still, it is uncertain for how long *Salmonella* will remain present at contamination levels >2 log CFU/g as only contamination levels ≤2 log CFU/g resulted in absence of ST at the end of experiments. Further, this study was based on a single contamination event while it is uncertain if a repetitious introduction of *Salmonella* might support longer persistence of *Salmonella* in the larvae even at low contaminations levels.

Wynants et al. (2019) found that *Salmonella* survived well in the wheat bran without larvae during the experimental period of 7 days. Further, the substrate without larvae had higher *Salmonella* counts or more *Salmonella*-positive replicates than substrate with larvae, which indicated that the larvae supported reduction of *Salmonella*. Contrary, our results for the contamination level of 5.4 log CFU/g indicated that ST counts in substrate were higher when larvae were present. Irrespective, at least no proliferation of *Salmonella* was observed in neither substrate nor larvae in both studies. For substrate, which is mainly dry, this may be explained by a required water activity *a*_w_ level of minimum 0.93 to facilitate growth of *Salmonella*, while *Salmonella* easily survive under dry conditions (Podolak et al., 2010). However, the humid rearing conditions for mealworms (often 50–70% RH) and addition of wet substrate as water source can be suspected to create ‘wet spots’ facilitating growth. No *a*_w_ measurements of the contaminated substrate were conducted in this study, but previous in-house measurements had indicated an *a*_w_ of 0.86 1 h after addition of spent grain in rearing boxes, i.e., well below the growth supportive level. So it is uncertain if the higher ST counts observed in substrate with larvae in our study were partly due to the continuous addition of spent grain increasing the moisture content, opposed to the single addition to trays without larvae to prevent molding. Further, as the gastrointestinal tract of the larvae provides humid conditions, it can be speculated if the passage of *Salmonella* through the larval gut is actually conducive for their survival after excretion considering the higher level of *Salmonella* found in substrate with larvae. The pH is 5.6 in the anterior and middle midgut of *T. molitor* larvae and 7.9 in the posterior midgut, i.e., there is no significant gastric acid barrier in the larvae acting on *Salmonella* that tolerate pH down to 4 (Moreira et al., 2017). This is in contrast to pH values as low as 2.0 and 3.1 in the middle midgut of larvae of black soldier flies (*Hermetica illucens*) and house flies (*Musca domestica*), respectively, assumingly reducing the change of *Salmonella* surviving passage through these fly larvae (Terra and Regel, 1995; Bonelli et al., 2019). Still, in *T. molitor* as in other insects, are antimicrobial peptides (AMP) reported to act in the defense against bacterial infections (Wu et al., 2018; Jo et al., 2019; Keshavarz et al., 2020). Nevertheless, Crippen et al. (2012) found that larvae of lesser mealworm excreted live *Salmonella* into their frass.

## Conclusion

Considering the long-time efforts made for optimizing and standardizing bacteriological culturing protocols for monitoring and safety control of other food and feed production chains, similar standardization of methods for monitoring of insect production or products would help to ensure validity and comparability of test results within this completely new area. Not least if specific hygiene process criteria or food safety criteria for edible insects are to be established in future.

This study indicated the importance of avoiding introduction of *Salmonella* into the mealworm production site, e.g., via contaminated substrate in order to avoid *Salmonella-*positive larvae as they remained positive for at least 14 days when the initial contamination levels were ≥3.4 log CFU/g. However, this study was based on a single contamination event and the impact of repetitious introduction of *Salmonella* should be assessed to neglect contaminations <2 log CFU/g. The initial contamination level affected the resulting *Salmonella* count in both larvae and substrate, and although the *Salmonella* level generally decreased over time and no proliferation of *Salmonella* was observed, proper treatment before consumption would be needed to ensure the food safety of larvae.

## Data Availability Statement

The raw data supporting the conclusions of this article will be made available by the authors, without undue reservation, to any qualified researcher.

## Author Contributions

AJ and SH contributed design and conductance of study and data analysis. AJ wrote the first draft of the manuscript and performed statistical analysis. DB and AJ contributed conception and management of the project. All authors contributed to discussion of results, manuscript revision, and read and approved the submitted version.

## Conflict of Interest

The authors declare that the research was conducted in the absence of any commercial or financial relationships that could be construed as a potential conflict of interest.
